# Novel Gradient-based Approach to Spatially Fractionated Radiotherapy: Implementation and Preliminary Clinical Results

**DOI:** 10.7759/cureus.91740

**Published:** 2025-09-06

**Authors:** Manuel Santos Ortega, Diana Guevara Barrera, Damian Guirado Llorente, Silvia Rodríguez Villalba, Francisco Blázquez Molina, Jose D Lago Martín, Jose Pérez-Calatayud

**Affiliations:** 1 Radiation Oncology, Hospital Clínica Benidorm, Benidorm, ESP; 2 Medical Physics, Hospital Universitario San Cecilio, Granada, ESP; 3 Medical Physics, Centro de Investigación Biomédica en Red de Epidemiología y Salud Pública (CIBER), Granada, ESP; 4 Medical Physics, Hospital Clínica Benidorm, Benidorm, ESP; 5 Medical Physics, Hospital Vithas Valencia Consuelo, Valencia, ESP

**Keywords:** bulky tumors, gradient-based radiotherapy, heterogeneous dose distribution, palliative, spatially fractionated radiation therapy

## Abstract

Background and purpose

Bulky tumors often present limited treatment options and poor response to conventional radiotherapy due to hypoxia, radioresistance, and dose delivery challenges near organs at risk. Spatially fractionated radiation therapy (SFRT) has shown promising results by creating heterogeneous intratumoral dose distributions that enhance local control while minimizing toxicity and stimulating immune response. We introduce a novel gradient-based approach, spatial stepwise gradient radiation therapy (2SG-RT), designed to simplify planning, preserve the biological advantages of dose heterogeneity, and facilitate integration into routine clinical workflows.

Materials and methods

Twenty-nine patients with pathologically confirmed malignant neoplasms manifesting as bulky tumors at various sites underwent 2SG-RT between November 2021 and April 2024. Target volumes were delineated in a stepwise manner, generating nested concentric planning target volumes (PTVs) for dose escalation, guided by PET-CT or CT-defined hypoxic/necrotic regions. Standard prescriptions were 12 Gy to the innermost PTV, 6-9 Gy to intermediate volumes, and 3-4 Gy to the outer PTV. Treatment planning employed intensity-modulated radiation therapy (IMRT) or volumetric modulated arc therapy (VMAT) to achieve the intended dose gradient while minimizing exposure to organs at risk, followed by conventional external beam radiation therapy (EBRT) with homogeneous dose.

Results

Among 29 patients (median age 70 years), median follow-up was four months. Median initial tumor volume was 118 cc. Median doses delivered with 2SG-RT were 12 Gy to the innermost PTV, 9 Gy to the intermediate PTV, and 3 Gy to the outer PTV. Radiologic assessment showed complete response in 34.5% of patients, partial response in 48.3%, and stability in 10.3%; two patients could not be assessed due to early death. Median tumor volume reduction was 75%. Symptom relief was complete in 44.8% and partial in 31% of patients. Treatment was well-tolerated with no grade 3-4 toxicities. Estimated equivalent uniform doses (EUD) and equivalent dose in 2 Gy fractions (EQD2) calculations indicated that the initial 2SG-RT fraction delivered, on average, a 106% higher biological dose than a single conventional fraction, while the full treatment regimens were comparable (mean difference 7.9%).

Conclusion

2SG-RT is a feasible and promising approach to SFRT, offering efficiency, adaptability, and applicability across diverse bulky tumors. It facilitates precise dose delivery, enhances planning and verification efficiency, and may support subsequent radiotherapy or systemic treatments in patients otherwise limited to palliative care. Preliminary clinical outcomes and EUD analyses suggest potential immune-mediated effects, warranting further investigation. Prospective comparative studies with longer follow-up are needed to validate these findings and clarify the clinical impact of 2SG-RT.

## Introduction

Bulky tumors challenge clinicians in daily practice. Often unresectable with limited treatment options, many patients receive palliative treatment or best supportive care [1]. Radiotherapy is an alternative, primarily for symptom control. Response varies in voluminous tumors due to factors like cell radioresistance, tumor hypoxia, or difficulty achieving adequate dose distribution in deep-seated tumors or near organs at risk (OAR) [2].

Spatially fractionated radiation therapy (SFRT) has emerged as an approach for large tumors. Derived from grid therapy used since the 1930s, it has achieved significant responses [3]. Its 3D evolution, lattice radiotherapy (LRT), was proposed in 2010 [4]. LRT creates intentional heterogeneity in intratumoral dose distribution, with geometrically spaced spherical volumes of ablative doses alternating with lower dose areas. This "peak-valley" distribution delivers higher doses to the tumor than conventional radiotherapy, minimizing toxicity while activating biological mechanisms promoting immune response and tumor suppression. LRT is proposed for various bulky tumors unsuitable for conventional external beam radiotherapy (EBRT) or stereotactic body radiation therapy (SBRT). It is considered a standard-of-care option for selected tumors, combined with conventional radiotherapy and sometimes systemic therapies [5].

Alternative approaches to SFRT have been proposed, such as stereotactic body radiotherapy - partial tumor irradiation targeting hypoxic segment (SBRT-PATHY), which targets only the tumor's hypoxic area, sparing the surrounding normoxic tumor and including circulating lymphocytes and peritumoral environment as OAR to activate the immune response [6]. These techniques, using high-dose gradients, modify treatment approaches in complex cases, presenting research opportunities for combining immuno-modulating agents with radiotherapy [1,7].

The complete elucidation of SFRT radiobiological pathways remains pending; however, radiation-induced bystander effects (RIBEs) have been documented [8] in unirradiated cells adjacent to target volumes. These effects are mediated via gap-junction intercellular communication or secretion of signaling molecules [2-4]. Additional phenomena, such as abscopal effects, intratumoral endothelial radiosensitization with larger radiation doses, and enhanced immunological responses due to intratumoral heterogeneity and tumor microenvironment alterations, have been described [3,4,9].

Although attempts have been made by Mayr et al. [10], Wu et al. [4], and Grams et al. [11] to standardize SFRT and LRT treatments by proposing implementation guidelines, currently, there is no directive addressing patient selection, optimal tumor volume, or technique. In its application, there exists considerable variability in sphere/vertex volume, sphere separation, and sphere placement in specific tumor areas. There is also a lack of consensus regarding sphere and valley dose, number and timing of LRT sessions, and combination with conventional radiotherapy. Certain bulky tumors may be too large for local control through conventional EBRT, yet not large enough to facilitate LRT plan design. Despite treatment variability and implementation challenges, as evidenced by Mayr et al. [5] and Iori et al. [2], clinical outcomes are frequently excellent, offering promise for complex bulky tumor treatment.

This study implements a novel technique for a broad spectrum of tumor presentations. This method is proposed as another alternative of SFRT, conceived to enhance efficiency and feasibility in daily clinical practice while maintaining dosimetric and biological advantages. Unlike established LRT techniques based on discrete vertices, this approach uses concentric stepwise dose-gradients, culminating in a conventional dose on the entire planning target volume (PTV). The primary objective of this study is to describe and present this gradient-based technique, which we have called spatial stepwise gradient radiation therapy (2SG-RT), while the secondary objective - reporting preliminary clinical results from its application in our center - builds directly on the first, illustrating its practical implementation and initial outcomes.

## Materials and methods

A cohort of 29 patients (20 men/nine women), with a median age of 70 years, received treatment at our center between November 2021 and April 2024. All subjects had histologically confirmed malignant neoplasms, manifesting as bulky masses in various anatomical locations. Diverse histological types were described (Table 1). Diagnostic computed tomography (CT) scans were performed on all patients, with 18 (62%) additionally undergoing 18-fluorodeoxyglucose (18-FDG) positron emission tomography (PET-CT) scans. The therapeutic approach was palliative for 16 patients (55.2%) and definitive for 13 patients (44.8%). Treatment targeted 16 primary lesions and 13 metastatic lesions. All patients receiving definitive treatment had locally advanced pulmonary malignancies, and in seven cases (24.1%), concurrent chemotherapy was administered.

**Table 1:** Tumor histologies and location Different tumor histologies observed in the series and anatomical locations of the treated lesions

Anatomical region involved	Number of patients (%)
Head and neck	7 (24.1)
Thorax (bone and soft tissue)	2 (6.8)
Thorax (lung)	15 (51.7)
Abdomen/pelvis	4 (13.8)
Extremities	1 (3.6)
Histology	Number of patients (%)
Squamous carcinoma	11 (37.9)
Adenocarcinoma	10 (34.5)
Hepatocarcinoma	2 (6.8)
Plasmacytoma	2 (6.8)
Large-cell carcinoma	1 (3.6)
Sarcoma	1 (3.6)
No information	2 (6.8)

Patient simulation and target volume delineation

All patients underwent a simulation CT with couch immobilization. The delineation process followed a stepwise approach. First, the gross tumor volume (GTV) was contoured on the planning CT. A uniform symmetrical expansion of 0.5-1 cm was then applied to generate the lowest-dose PTV, which encompassed the entire GTV and accounted for setup uncertainties and internal organ motion. This lowest-dose PTV was not modified to spare adjacent OAR, as it corresponded to a more conventional prescription dose.

Within this PTV, additional nested volumes were created to permit dose escalation. The innermost PTV, designated to receive the highest dose, was delineated by direct fusion with PET-CT imaging when available, with regions of hypermetabolic activity selected if they exhibited more than 10% metabolism above the median standardized uptake value (SUV) of the tumor, thereby including areas of increased metabolic activity. In patients without PET-CT, clearly identifiable hypoxic or necrotic areas on CT were targeted for inclusion in the innermost PTV, used as surrogates for biological target volume definition.

**Figure 1:**
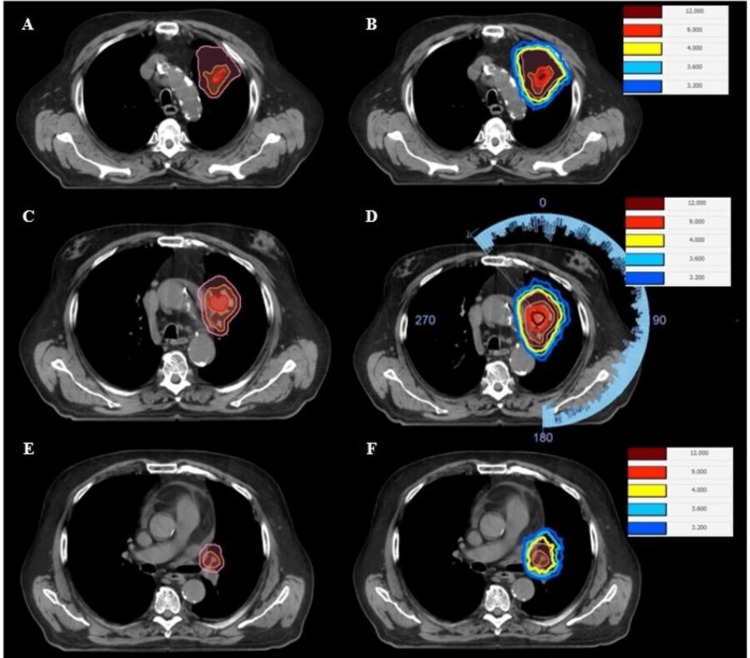
Representative case illustrating volume generation and dosimetric planning methodology in spatial stepwise gradient radiation therapy (2SG-RT) for a patient treated for lung carcinoma. Axial planes are shown at three levels: A,B: 1 cm cranial to the gross tumor volume (GTV); C,D: mid-GTV; E,F: 1 cm caudal to the GTV. Panels A, C, and E show volume placement, while panels B, D, and F show the corresponding isodose distribution. Isodose curves: maroon: 12 Gy, red: 9 Gy, yellow: 4 Gy, light blue: 3.6 Gy, dark blue: 3.2 Gy.

The intermediate-dose PTV was subsequently generated as a concentric shell around the innermost PTV, applying an outer symmetrical margin, usually approximately 1 cm from this internal volume, but highly variable depending on tumor volume and shape, as well as the distance from the innermost PTV to the margin of the GTV. Figure 1 illustrates the delineation of these nested volumes in a recently treated lung patient, providing a representative example of the stepwise contouring strategy.

Standard dose prescriptions were 12 Gy for the innermost PTV and 6-9 Gy for the intermediate PTV, depending on the distance from the highest dose region and the requirement to maintain dose conformity in the outer PTV (generally 3-4 Gy, not exceeding 5 Gy). In our series of 29 patients, only two required delineation of a single concentric PTV due to tumor size or femoral involvement.

Treatment planning and delivery

Two radiation therapy plans were generated: one for 2SG-RT, administered on the first day and a second for conventional EBRT, with a homogeneous dose in the entire PTV. Treatments were administered consecutively. Intensity-modulated radiation therapy (IMRT) or volumetric arc therapy (VMAT) was used or 2SG-RT planning. For conventional EBRT (second phase), three-dimensional radiotherapy (3DRT), IMRT, or VMAT were employed. Since 2023, VMAT has been systematically implemented for the second-phase conventional EBRT. Treatment planning systems used were TPS Eclipse (Varian Medical Systems, Palo Alto, CA, USA) version 11.0 and Monaco (Elekta AB, Stockholm, Sweden) version 6.1.3.0. IMRT was used with Eclipse TPS to achieve the required gradient in inner volumes. With Monaco TPS, VMAT became the preferred technique.

Below is a brief description of the dosimetric planning methodology for the representative case of the lung considered in the description of volume generation.

VMAT arcs with a flattening filter-free (FFF) beam at maximum dose rate are used (with 6 MV energy, except in deep abdominal lesions without neighboring heterogeneous structures, in which case 10 MV is employed). The angular range of the arcs is wide enough to achieve the target concentric heterogeneity. Figure 1 illustrates an example of this range. Optimization prioritizes coverage of at least the median volume of 12 Gy, while shaping the lowest dose volume as much as possible. The dose to the intermediate auxiliary volume depends on the two aforementioned conditions. The aim is that, outside the PTV, the dose distribution in OAR and healthy tissue is modified as little as possible as a result of the dose escalation, thereby allowing subsequent phases to be carried out safely.

**Figure 2:**
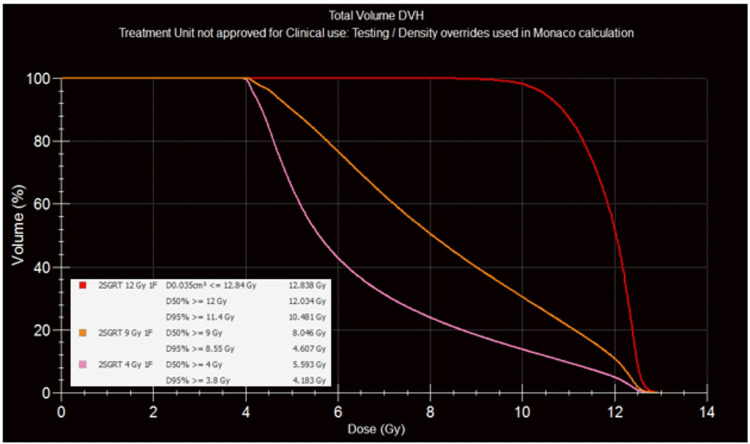
Dose-volume histograms for the representative case. Curves are shown for 12 Gy (red), 9 Gy (orange), and 4 Gy (pink). The inset displays the numerical values for the global maximum, 50% and 95%.

**Figure 3:**
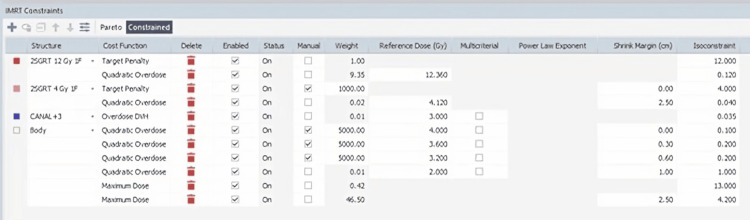
Example of typical optimization parameter selection in the Monaco TPS for spatial stepwise gradient radiation therapy (2SG-RT) planning Monaco TPS was used with Monte Carlo algorithm, applying the cost function, weights, reference doses, margins, and isoconstraints.

Figure 1 depicts a representative case with contouring and dose distribution in multiple cross-sections, while Figure 2 shows the dose-volume histograms for 12 Gy, 9 Gy, and 4 Gy. In this recent case, the Monaco TPS was used with its Monte Carlo algorithm, applying the cost function, weights, reference doses, margins, and isoconstraints described in Figure 3. The aim is to use a calculation grid resolution of no more than 0.3 cm and a statistical uncertainty of 1%-3%.

Within Monaco TPS, the VMAT sequencing parameters are: segment shape optimization on, high precision leaf positions on, maximum number of arcs four, maximum number of control points per arc 180, minimum segment width 0.5 cm, fluence smoothing medium, and beamlet width 0.3 cm (within IMRT prescription parameters). The constrained optimization algorithm is applied.

Before the treatment, all 2SG-RT plans are verified, as with all VMAT plans, using Octavius4D (PTW, Freiburg, Germany) with the 1500 or 1600 array, with inter-detector distances of 0.25 cm and 0.71 cm, respectively, depending on the irradiation volume size. For PTVs smaller than 15 cm in any direction, the 1600 array is used. The plans are evaluated using the VeriSoft v.8.1 package (PTW, Freiburg, Germany). Tolerance limits and methodologies for verification are based on the American Association of Physicists in Medicine (AAPM) TG-218 report [12].

Planning aimed to attain the prescribed higher dose while maintaining PTV conformation and OAR constraint compliance. OAR doses remained within tolerances for the entire treatment. In our department, the constraint values published by Timmerman [13] are taken as reference.

Daily image guidance primarily used cone-beam CT (CBCT). Adaptive radiotherapy was implemented for significant tumor response during conventional EBRT. The criteria for adaptation are highly dependent on each individual case, as determined by the interdisciplinary tumor board, and are based primarily on the percentage of tumor volume reduction observed in the follow-up CT scan.

Follow-up

Patients were followed during radiotherapy to assess clinical response and potential toxicities, and later every three to six months when possible.

Radiological response was assessed using Response Evaluation Criteria in Solid Tumors (RECIST) V1.1 [14], and metabolic response with PET response criteria in solid tumors (PERCIST) V1.0 [15]. The remaining tumor was contoured on response evaluation imaging to calculate residual volume. Toxicities were analyzed using Common Terminology Criteria for Adverse Events (CTCAE) V5.0 [16].

Radiobiological analysis of the effect of heterogeneity in dose distribution

Although the radiobiology involved in SFRT is complex, this section attempts to evaluate the effect of dose heterogeneity using established models, with the sole purpose of providing an approximate estimation.

Equivalent uniform dose (EUD) is the absorbed dose that, homogeneously distributed in the volume of interest, produces the same effect as the real heterogeneous distribution, and can be used for the analysis and comparison of treatments [17]. Considering survival as the final effect:

$\operatorname{SF}\left(EUD)\right) = \overline{SF}$ (1)

where *SF *is the mean survival fraction in the volume of interest produced by the actual dose distribution, and *SF(EUD)* is the survival produced by the *EUD* uniformly distributed in that same volume. If there is a dose-volume histogram of the tissue of interest provided by the planning system with *m* intervals, the equation can be written:

$\operatorname{}N\cdot\overline{SF}=\sum_{\mathrm{i}=1}^{\mathrm{m}}N_{\mathrm{i}}\cdot {SF}_{\rm i}=\sum_{\mathrm{i}=1}^{\mathrm{m}}{\frac{V_{\rm i}}{V}}\cdot N \cdot{SF}_{\rm i}$ (2)

where *N* and *V* are the total number of cells and the total volume of the tissue, respectively, and *N_i_*, *V_i_*  and *SF_i_* the cell number, volume and survival corresponding to the *i*-th bin of the histogram, respectively, assuming identical cell density throughout the tissue. So that:

$\overline{SF}=\sum_{\mathrm{i}=1}^{\mathrm{m}}\nu_{\mathrm{i}}\cdot{SF}_{\rm i}$ (3)

with $\nu$ the i-th volume fraction. From the linear quadratic (LQ) survival model:

$\operatorname{}SF\left(d\right)\equiv SF=\exp\left[-\alpha\cdot d-\beta \cdot d^2\right]$ (4)

where $\alpha$ and $\beta$ are the parameters of the LQ model, and *d* is the absorbed dose.

For comparing treatments utilizing the newly developed 2SG-RT technique and the conventional schedule, the histograms of each treatment phase are employed to calculate the corresponding EUD per fraction. It is feasible to calculate the total equivalent dose to that administered in fractions of 2 Gy EQD2 for each treatment type. Through this method, the doses of each phase of the 2SG-RT treatment can be added and compared with the total equivalent dose of the conventional treatment.

Thus, for conventional treatment with *n* fractions:

$\operatorname{}EQD2_{\rm c}=n \cdot {EUD}_{\rm c}\cdot \frac{EUD_{\rm c}+\alpha / \beta}{2+\alpha / \beta}$ (5)

And for 2SG-RT treatment:

$\operatorname{}EQD2_{\rm {2SG}}={EUD}_{\rm {2SG}}\cdot \frac{EUD_{\rm {2SG}}+\alpha / \beta}{2+\alpha / \beta}+\left( n- 1\right)\cdot{EUD}_{\rm c}\cdot \frac{EUD_{\rm c}+\alpha / \beta}{2+\alpha / \beta}$ (6)

The parameter values utilized to perform the calculations are $\alpha=0.3 \mathrm{Gy}^{-1}$, $\beta=0.03 \mathrm{Gy}^{-2}$ and $\alpha/\beta =10 \mathrm{Gy}$. To estimate the uncertainties, Monte Carlo methods were employed, assuming that the parameter values can vary by 30% with respect to the aforementioned mean values according to normal distributions. Uncertainties are expressed with a coverage factor *k*=1.

## Results

**Figure 4:**
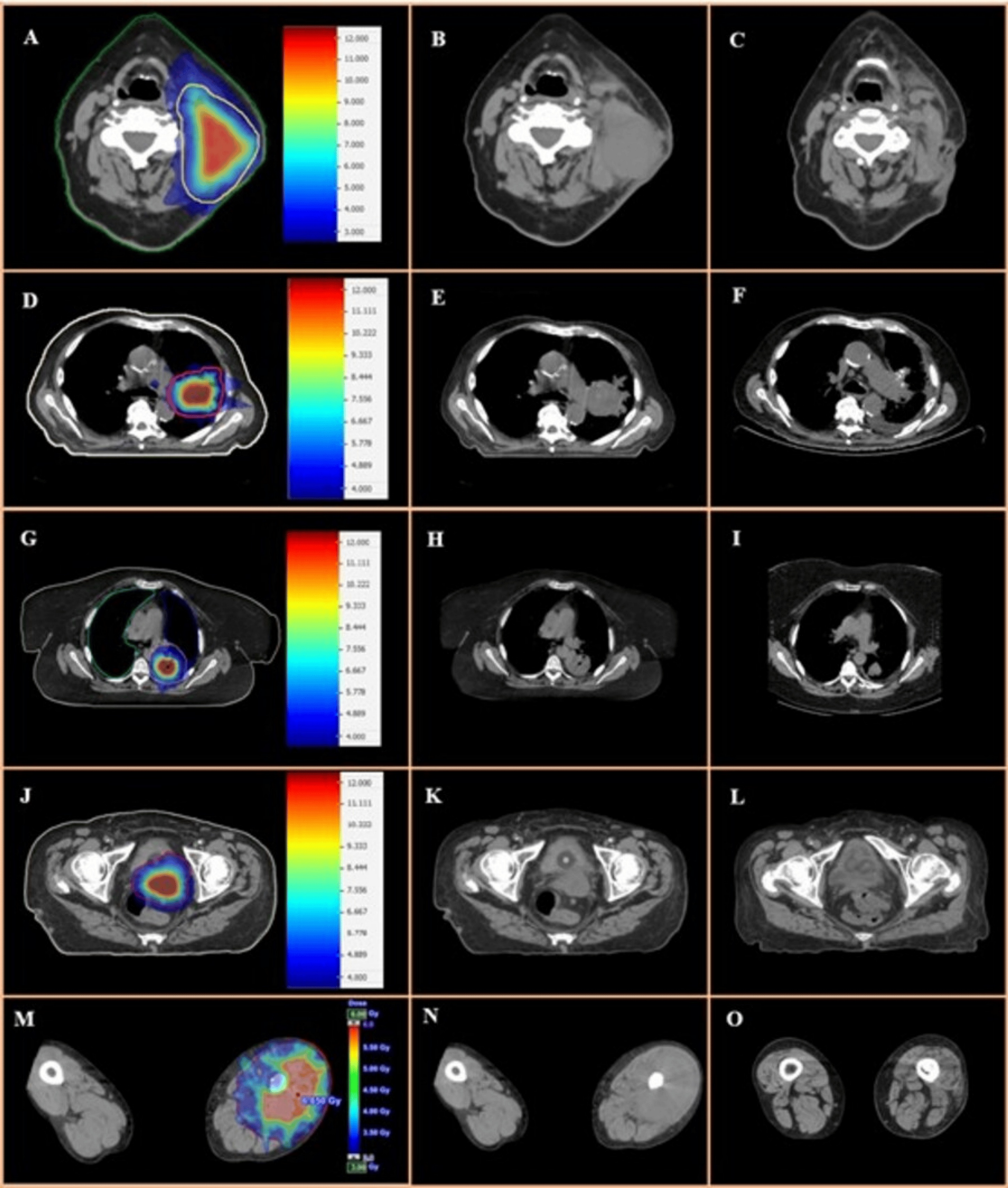
Examples of dose distributions for 2SG-RT and responses observed post treatment (2SG-RT and conventional EBRT). A-C: left cervical lymph-node metastasis from squamous carcinoma (cervical occult primary); A: dosimetry for 2SG-RT, B: diagnostic CT scan, C: CT scan at 2 months post-treatment. D-F: left lung primary adenocarcinoma (hilar lesion); D: dosimetry for 2SG-RT, E: diagnostic CT scan, F: CT scan at two months post-treatment. G-I: left lower lobe (lung) primary squamous carcinoma; G: dosimetry for 2SG-RT, H: diagnostic CT scan, I: CT scan at two months post-treatment. J-L: primary uterine sarcoma; J: dosimetry for 2SG-RT, K: diagnostic CT scan, L: CT scan at two months post-treatment. M-O: right lower limb metastasis from lung adenocarcinoma; M: dosimetry for 2SG-RT, N: diagnostic CT scan, O: CT scan at two months post-treatment.

The median age was 70 years (range, 48-88 years). The median follow-up was four months (range, 0-29 months). The median initial tumor volume was 118 cc (range, 48-1500 cc). The median dose to PTV, intermediate volume, and inner volume in 2SG-RT were 3 Gy (range, 2-5 Gy), 9 Gy (range, 4-9 Gy), and 12 Gy (range, 6-12 Gy), respectively. Complete radiologic response was observed in 10 patients (34.5%), partial response in 14 patients (48.3%), and radiologic stability in three patients (10.3%), with two of the latter having only one month of follow-up. In two patients (6.9%), response could not be assessed due to intercurrent death occurring shortly after radiotherapy. The median percentage of volume reduction (in assessable patients) was 75% (0-100) (Table 2). Thirteen patients (44.8%) experienced complete resolution of symptoms reported at the start of radiotherapy, and nine (31%) experienced partial resolution. One patient (3.4%) could not be assessed due to early intercurrent death. Six patients (20.8%) did not report any symptoms directly associated with the bulky tumor. The inhomogeneous treatment was well-tolerated by all patients, including those receiving concurrent systemic treatment. No G3-4 toxicities directly related to 2SG-RT were reported. All observed toxicities are detailed in Table 3. Figure 4 depicts dosimetry distributions and treatment responses in a subset of patients. 

**Table 2:** Tumor / treatment characteristics and outcomes Specific tumor characteristics for each case, and clinical / radiological responses evidenced after 2SG-RT and subsequent conventional EBRT. Pr+ = primary, M++ = metastasis, m+++ = months, CT± = chemoradiotherapy, C++++ = complete, P* = partial, N** = no, Y*** = yes, NA****= non-assessable, LLL- = left lower lobe, RUL> = right upper lobe, RLL< = right lower lobe, LULº = left upper lobe.

Patient	Anatomical location	Pr^+^ / M^++^	Histology	Concurrent CT^±^	Initial GTV volume (cc)	PTV dose (Gy)	Intermediate volume dose (Gy)	Inner volume dose (Gy)	Follow-up (m^+++^)	Symptom response	Radiological response	% of response	Residual tumor volume (cc)
1	Right cheek mucosa	M	Squamous	N^**^	100	3	9	12	6	C^++++^	C	100	0
2	Pelvis	M	No information	N	809	3	9	12	3	P^*^	S	0	780
3	Sternum	M	Plasmacytoma	N	786	3	9	12	1	P	P	40	470
4	Lung (LLL^-^)	Pr	Squamous	N	48	2,25	9	11,25	27	-	C	100	0
5	Left supraclavicular lymph node	M	Adenocarcinoma	N	87	3	9	12	4	P	P	54	40
6	Lung (RUL^>^)	Pr	Squamous	Y^***^	189	2,25	9	12	27	-	P	80	37
7	Lung (RLL^<^)	Pr	No information	N	104	3	9	12	10	-	S	0	100
8	Lung (LULº)	Pr	Adenocarcinoma	Y	192	2	9	12	0	P	NA^****^	NA	NA
9	Right cervical lymph node	M	Squamous	N	48	2,75	6	12	21	P	C	100	0
10	Right iliac bone	M	Plasmacytoma	N	876	2	4	6	15	C	C	100	0
11	Right Vastus Intermedius muscle	M	Large-cell	N	1500	3	6	-	29	C	C	100	0
12	Left cervical lymph node	M	Adenocarcinoma	N	135	2,25	6	10	5	C	C	100	0
13	Lung (RUL)	Pr	Adenocarcinoma	Y	269	3	9	12	21	C	P	74	70
14	Left scapula	M	Hepatocarcinoma	N	91	5	8	12	4	C	P	70	27
15	Lung (LUL)	Pr	Adenocarcinoma	N	71	2,25	6	-	9	P	P	55	32
16	Left supraclavicular lymph node	M	Hepatocarcinoma	N	101	5	9	12	0	NA	NA	NA	NA
17	Lung (LLL)	Pr	Squamous	Y	118	2,25	8	12	11	C	C	100	0
18	Lung (LUL)	Pr	Squamous	N	150	4	9	12	12	C	C	100	0
19	Left cervical lymph node	M	Squamous	N	190	3	9	12	8	C	C	100	0
20	Lung (RUL)	Pr	Adenocarcinoma	N	50	4	7	12	4	-	P	48	26
21	Left cervical lymph node	M	Squamous	Y	295	3	9	12	1	P	S	NA	NA
22	Lung (RLL)	Pr	Squamous	N	205	2,25	7	12	2	-	P	76	49
23	Vaginal vault	Pr	Adenocarcinoma	N	100	3	6	12	2	C	C	100	0
24	Lung (RUL)	Pr	Adenocarcinoma	N	88	4	9	12	2	P	P	62	33
25	Lung (RLL)	Pr	Adenocarcinoma	Y	184	2,25	4	9	1	P	P	63	68
26	Uterus	M	Sarcoma	N	215	4	6	12	1	C	P	49	110
27	Lung (RUL)	Pr	Squamous	N	61	4	8	12	1	-	P	92	5
28	Lung (LLL)	Pr	Adenocarcinoma	Y	73	4	9	12	0	C	P	65	25
29	Lung (LUL)	Pr	Squamous	N	67	3	4	9	0	C	p	64	24

**Table 3:** Reported toxicities Reported toxicities associated with 2SG-RT treatment LLL- = left lower lobe, RUL> = right upper lobe, RLL< = right lower lobe, LULº = left upper lobe.

Patient	Treatment location	Toxicity	Grade
1	Right cheek mucosa	Oral mucositis	2
4	Lung (LLL-)	Chest wall pain	1
7	Lung (RLL	Chest wall pain	1
7	Lung (RLL)	Pneumonitis	1
10	Right iliac bone	Diarrhea	2
11	Right Vastus Intermedius muscle	Leg lymphedema	2
12	Left cervical lymph node	Dermatitis	1
13	Lung (RUL>)	Esophagitis	2
15	Lung (LULº)	Dyspnea	2
16	Left supraclavicular lymph node	Dermatitis	1
19	Left cervical lymph node	Dermatitis	1
21	Left cervical lymph node	Dermatitis	2
21	Left cervical lymph node	Pharyngeal mucositis	2
23	Vaginal vault	Urinary urgency	2
26	Uterus	Urinary urgency	2
28	Lung (LLL)	Esophagitis	2

The estimated EUD values for each patient and each treatment regimen are presented in Table 4. The EQD2 corresponding to the initial fraction of the 2SG-RT regimen and to a single fraction of the conventional regimen (which would have been administered as an alternative to 2SG-RT) has been calculated, as well as their relative difference in percentage. This difference exhibits a mean value of 106%, with minimum and maximum values of 14% and 230%, respectively. These values have also been calculated for the complete treatment of the 2SG-RT regimen and for the full conventional treatment; in this instance, the mean relative difference is 7.9%, with a minimum value of 0.7% and a maximum value of 17.3%.

**Table 4:** EUD calculation results Comparison of EQD2 values per fraction and total treatment between the 2SG-RT technique and the conventional schedule across 29 cases. The table presents the number of fractions per case, mean EQD2 per fraction, total EQD2, and the percentage difference between techniques for both individual fractions (Δ1fr) and the entire treatment course (Δtotal).

Case	Fractions (dose)	EQD2_2SG-RT_ (Gy) (1fr)	EQD2c (Gy) (1fr)	Δ1fr (%)	EQD2_2SG-RT_ (Gy)	EQD2c (Gy)	Δtotal (%)
1	1+9	8.7±0.2	3.38±0.08	157±9	39.1±0.8	33.8±0.8	15.7±3.6
2	1+9	8.4±0.2	3.37±0.08	149±8	38.7±0.8	33.7±0.8	14.9±3.6
3	1+9	7.7±0.2	3.27±0.08	135±8	37.1±0.7	32.7±0.8	13.5±3.6
4*	1+19	6.1±0.1	2.31±0.06	162±9	50.0±1.1	46.2±1.1	8.1±3.6
5	1+9	5.6±0.1	3.24±0.08	73±6	34.8±0.7	32.4±0.8	7.3±3.4
6*	1+19	5.0±0.1	2.35±0.06	115±7	49.7±1.1	47.0±1.2	5.7±3.6
7	1+9	8.6±0.2	3.16±0.08	173±11	37.1±0.9	31.6±1.0	17.3±4.7
8	1+19	4.8±0.1	2.05±0.05	134±8	43.7±1.0	41.0±1.0	6.7±3.6
9	1+12	5.0±0.1	2.30±0.06	116±7	32.5±0.7	29.8±0.7	8.9±3.6
10	1+19	3.22±0.08	2.00±0.05	61±6	41.2±1.0	40±1.0	3.1±3.5
11	1+8	4.3±0.1	3.23±0.08	34±5	30.2±0.6	29±0.7	3.8±3.3
12*	1+14	4.2±0.1	2.30±0.06	83±6	36.4±0.8	34.5±0.9	5.5±3.7
13*	1+19	2.63±0.06	2.30±0.06	14±4	46.3±1.1	46.0±1.2	0.7±3.5
14	1+4	7.5±0.2	6.2 ±0.2	20±4	32.4±0.6	31.2±0.8	3.9±3.2
15	1+19	2.8±0.1	2.30±0.06	20±4	46.5±1.1	46.0±1.2	1.0±3.6
16	1+4	8.2±0.2	6.5±0.2	26±4	34.1±0.6	32.4±0.8	5.3±3.1
17	1+19	7.6±0.2	2.30±0.06	230±12	51.3±1.2	46.0±1.2	11.5±3.8
18	1+9	8.2±0.2	3.25±0.08	151±8	37.4±0.7	32.5±0.8	15.1±3.6
19	1+9	6.8±0.2	3.24±0.08	110±7	36.0±0.7	32.4±0.8	11.0±3.5
20	1+19	4.3±0.1	2.30±0.06	88±7	48.0±1.1	46.0±1.2	4.4±3.7
21*	1+27	5.3±0.1	1.77±0.05	200±11	53.1±1.4	49.6±1.4	7.1±4.1
22	1+19	4.7±0.1	2.30±0.06	106±7	48.4±1.1	46.0±1.2	5.3±3.7
23	1+9	7.5±0.2	3.25±0.08	131±8	36.7±0.7	32.5±0.8	13.0±3.6
24*	1+19	6.7±0.2	2.30±0.06	190±10	50.4±1.2	46.0±1.2	9.5±3.8
25*	1+19	3.58±0.09	2.30±0.06	56±6	47.3±1.1	46.0±1.2	2.8±3.7
26	1+9	6.1±0.1	3.25±0.08	89±6	35.4±0.7	32.5±0.8	8.9±3.5
27	1+9	6.6±0.2	3.25±0.08	104±7	35.9±0.7	32.5±0.8	10.4±3.5
28*	1+19	4.2±0.1	2.30±0.06	84±7	47.9±1.1	46.0±1.2	4.2±3.7
29	1+9	4.9±0.1	3.25±0.08	52±5	34.2±0.7	32.5±0.8	5.2±3.4
* These patients were given a third phase of treatment in a different volume (PTV)							

## Discussion

LRT is being utilized for treating bulky tumors, showing favorable clinical and imaging responses. The underlying biological mechanisms remain under investigation, although bystander effects have been demonstrated in vitro and in vivo. Factors like potential correlation between treatment response and dose heterogeneity are beginning to be addressed in recent literature [5,9]. Given the technique's relative novelty, a standardized approach has not been established, resulting in variation in prescription doses and lattice array design strategies [2]. This single-institution, retrospective feasibility study reports early clinical results of 2SG-RT, focusing on practical implementation rather than comparative efficacy.

We propose an alternative approach to SFRT for complex bulky tumors. Our department has extensive experience treating tumors with internal volume dose intensification. We adapt both concepts to create inner areas of dose intensification in a stepwise manner, with an internal ablative dose volume, an external PTV with a conventional treatment dose and, in most patients, an intermediate dose volume. Challenges in LRT include sphere placement, planning complexity, and increased duration for optimization, verification, and delivery. In our experience, LRT required considerably more time for treatment planning and delivery. As for treatment delivery, the duration of a 2SG-RT session is comparable to that of a conventional VMAT radiotherapy session. In our appreciation, using 2SG-RT, the reduction in contouring and dosimetric planning time exceeded a factor of five. The primary challenge with LRT in our modest experience was deciding on sphere placement, which varied greatly from case to case. In contrast, 2SG-RT demonstrates enhanced adaptability and efficiency. This approach considers tumor biological characteristics, facilitates uniformity and predetermined objectives, augmented by CT and PET-CT for placement guidance.

Regarding potential inter-planning variability in 2SG-RT application, we believe that planning demonstrates an adequate level of robustness, as an optimization template is applied to all patients with only minor modifications needed for individual cases.

This strategy was implemented for patients with indications for palliative radiotherapy or locally advanced bulky lung tumors unable to receive curative doses. The heterogeneous 2SG-RT plan was administered on the initial day, consistent with LRT, to apply the same concept (immune response and bystander effects activation). The highest tumor dose was selected to trigger immune response and biological effects while allowing conventional PTV dose and meeting OAR constraints. The inner-volume dose was frequently 12 Gy, comparable to LRT vertex dose in previous studies [18-20]. In one patient, the highest inner-dose volume reached only 6 Gy, constrained by OAR proximity, in a radiosensitive histology (plasmacytoma).

More than three "steps" or dose volumes within the tumor were not utilized due to limited tumor volume. Inner/ablative dose volumes were delineated based on areas of maximum activity in PET-CT, when available, as this method targets the most biologically active tumor regions and ensures non-tumor tissues are not included in this higher-dose volume [21]. In cases without PET-CT, hypointensities in CT were targeted, indicating radioresistant areas within the tumor. Optimal timing of lattice-type treatments relative to EBRT is not well defined, but it is generally accepted to be within the first five days [18-20]. We have aimed to follow this guideline, administering 2SG-RT on day one and consecutively following with EBRT. In certain cases, due to various factors and departmental logistics (e.g., following a weekend), EBRT was delayed up to 48 hours. This gap reflects adaptations made to align treatment with practical considerations while maintaining the overall treatment strategy.

LRT has typically been administered on day one of treatment, although some investigators have administered multiple LRT fractions either as the sole therapeutic modality or followed by conventional EBRT [18-20, 22]. After heterogeneous or partial tumor irradiation at high doses, immune response can be activated, which requires a window of time that is not precisely defined. In our approach, this is taken into account when scheduling EBRT after 2SG-RT, aiming to optimize the potential biological effects while adhering to practical logistics. Optimizing the timing of SFRT with potential systemic therapies and subsequent conventional radiation is essential and under investigation [23].

Our preliminary results show favorable outcomes in follow-up imaging and symptom control, aligning with findings in LRT series and SBRT-PATHY [6] or the stereotactic central/core ablative radiation therapy (SCART) technique [24]. These results reflect early clinical experience and should be interpreted in the context of a single-institution feasibility study, not as evidence of comparative efficacy. Previously published LRT works are summarized in Table 5. The series varies greatly in patient numbers (1-50), histologies, primary tumor location, and complete responses (30-84%).

**Table 5:** Previously published works in LRT Previously published series and studies in Lattice radiotherapy. CBCT: Cone-beam CT; CR: complete response; CTC: center-to-center; DFSS: disease-free specific survival; EBRT: External beam radiotherapy; fx: fractions; G: grade; GTV: gross tumor volume; LC: local control; LRT: lattice radiotherapy; LVD: lattice volume dose; MFU:  median follow-up; MRI: magnetic resonance imaging; NS: not specified; NSCLC: non-small cell lung cancer; OS: overall survival; PR: partial response; PTV: planning target volume; RT: radiotherapy; FIGO: Fédération Internationale de Gynécologie et d'Obstétrique.

Author	Number of patients (n)	Tumor type / histology	RT dose / fractionation	Initial GTV volume	Vertex diameter	Toxicity	Response
Schiff et al. [18] (2021)	1	Endometrial Adenocarcinoma FIGO IVB	5 fx LRT PTV dose 4 Gy/fx LVD 13 Gy/fx	625 cc	1.5 cm. CTC spacing 4 cm	Tumor lysis syndrome and death.	28% volume reduction at two weeks after completion of RT
Price et al. [19] (2023)	2 Follow-up Patient 1: 15 months Patient 2: 6 months	Patient 1: liver metastasis from primary Leiomyosarcoma Patient 2: liver metastasis from primary cholangiocarcinoma. *MRI-guided	5 fx LRT: PTV dose 4 Gy/fx LVD 13 Gy/fx	Patient 1: 170.4 cc Patient 2: 302.7 cc	1.5 cm	NS	At 3 months Patient 1: 87% volume reduction Patient 2: 14.6% volume reduction. Both patients later progressed locally, regionally, and distantly
Duriseti et al. [20] (2022)	20 Follow-up 3 months	-Soft tissue sarcomas 45% -NSCLC 35% -Thymic carcinoma 5% -Malignant mesothelioma 5% -Endometrial adenocarcinoma 5% -Colonic adenocarcinoma 5%	5 fx LRT: PTV 4 Gy/fx LVD 13 Gy/fx	Median 579.2 cc Maximum 3713 cc	1.5 cm	One patient G2 pneumonitis One patient G2 diarrhea, G3 Transaminitis, and G4 urosepsis, requiring pressor support	At a median of 81 days: Median 24.4% volume shrinkage in GTV 36% of evaluable tumors with 80% volume reduction at 3-6 months
Amendola et al. [22] (2020)	10 MFU 16 months (1-77)	Cervix FIGO stage IIIB-IVA	3 fx LRT: PTV dose 3 Gy/fx LVD 8 Gy/fx Subsequently EBRT mean dose 44.28 Gy Mean BED: LVD: 95.45 Gy Tumor: 79.54 Gy	Mean 200.35 cc	1-1.5 cm	Acute: G1 diarrhea 1 G2 cystitis (bladder involvement with tumor) No G3-4	Mean volume reduction during treatment 54% Early CR 88.9% and PR 11.1% 2-year LC 100% DFSS 53.3%
Ferini et al [25] (2022)	30 (31 treatments) MFU 10.75 months	Multiple sites (4 head and neck, 5 intrathoracic, 15 abdomen-pelvis, 2 breast, 4 soft-tissue, 1 lower extremity)	1-3 fx LRT: PTV dose NS LVD 7-18 Gy/fx *Lattice sphere placement guided by PET CT. Subsequent EBRT median 20 Gy in 4 fx	Median 146.48 cc (50.9 – 2039.7)	1 cm. CTC spacing 2 cm	No G3-4 acute toxicities	Complete clinical response 11 patients Partial clinical response 4 patients
Ahmed et al [26] (2023)	53 (61 treatments) 9 treatments with brass and proton GRID	Sarcomas (46 soft-tissue, 15 bone). Diverse anatomical locations	1 fx LRT: PTV dose NS LVD 16-20 Gy Subsequently EBRT median dose 40 Gy in a median of 10 fx	Median 636 cc (47 – 13373)	NS	G3-4 in 4 patients (1 skin, 2 GI, 1 pneumonitis)	At median of 6 months: 35% stable imaging 56% PR 10% progression 1-year LC 82% 1-year OS 53%
Amendola et al [27](2019)	10 MFU 6 months (1-71)	NSCLC	1 fx LRT: PTV dose 3 Gy LVD: 18 Gy Subsequently EBRT 25 - 33 fx 1,8- 2 Gy	Mean 195 cc	0,8-1.5 cm. Mean CTC spacing 3,6 cm.	100% G1 radiation pneumonitis in FDG- PET-CT	Median 64% tumor reduction in CBCT of last day RT. Median tumor response at a median of 6 months follow-up 43% (10-53%).
Larrea et al [28](2020)	9 Follow-up 2 weeks	Lung (n=5) Axillary sarcoma (n=1) Cervix (n=1) Head and neck (n=1)	1 fx LRT: PTV dose 2 - 3.5 Gy LVD 15 - 18 Gy	Median 180,2 cc	1 cm. CTC spacing 2,5 - 3 cm	NS	2 patients > 50% tumor reduction 2 weeks after LRT
Borzov et al [29] (2022)	3 MFU NS	Soft tissue sarcomas All in the hip region.	1 fx LRT: PTV dose 2.5 - 4 Gy LDV 20 Gy Subsequent EBRT 50 Gy in 25 fx Subsequent resection	NS	Cilinders 1cm height x 1cm diameter. Spacing 1-2cm	1 patient with skin healing problems after resection.	2 patients with complete pathological response at surgery. 1 patient with at least 3mm clean resection margins
Dincer et al [30] (2022)	2	2 liver metastases from squamous anal canal tumor, and rectal adenocarcinoma	5 fx LRT: PTV dose 6 Gy/fx LVD 10 Gy/fx	Case 1: NS Case 2: 494 cc	NS	No G3-4	Case 1: near complete regression in PET CT at three months after LRT. Case 2: 53% volume reduction one month after LRT.
Prado et al [31] (2024)	10	4 abdominal sarcomas 1 lung 2 liver 1 mediastinum 2 node metastases	EBRT ranging from 20 – 40 Gy in 5 – 20 fx. Subsequently 1 fx LRT: PTV dose 4-5 Gy LVD 18 Gy	Mean 1637 cc (416.7 – 3614.6)	1.5 cm CTC spacing 2.5 – 3 cm	NS	NS
Xu et al [32] (2024)	19 Follow-up: 3 months	Head and neck carcinomas *6 previous EBRT	1 fx LRT: PTV dose 4 Gy LVD 12 Gy	Mean 208 cc (48 – 701)	0.4 cm CTC spacing 2 cm	No G3-4 reported G2 mucositis (n=3) G1 dermatitis (n=2) G2 dysphagia (n=1)	At 1 month: Tumor regression 84% 3 with regression >75%

Tolerance to our 2SG-RT approach was excellent, with no Grade 3-4 toxicity reported. Previous SFRT studies noted toxicities including: Grade 2 mucositis and pneumonitis (Ferini et al. [25], Duriseti et al. [20]); Grade 4 urosepsis and Grade 3 transaminitis in one patient (Duriseti et al. [20]); Grade 3-4 toxicity in 8% of patients (Ahmed et al. [26]); and a fatal tumor lysis syndrome in an 85-year-old patient treated with LRT for FIGO stage IVB clear cell endometrial carcinoma (Schiff et al. [18]). This patient received LRT in five fractions, with 20 Gy PTV total dose and 66.70 Gy vertex total dose. Despite 28% tumor volume reduction two weeks post-radiotherapy, tumor lysis syndrome occurred 13 days after treatment. Risk-stratification for TLS is recommended before LRT, especially in patients with radiosensitive tumors or preexisting comorbidities.

Using EQD2 calculations, we compared this novel treatment strategy against theoretical conventional treatment for each case. Our findings show a modest increase in EQD2 for 2SG-RT treatment relative to conventional treatment of 7.9% on average, presenting challenges in explaining the favorable clinical outcomes observed for many patients. Consequently, the observed clinical outcome may be attributed to the effect of the initial dose fraction, speculatively related to immune-mediated mechanisms induced by treatment heterogeneity rather than solely by dose escalation, including bystander and abscopal effects, intratumoral endothelial radiosensitization, and enhanced immunological responses.

We aimed to characterize intensification through EUD, acknowledging that the alpha/beta model may not fully account for the observed clinical effects, but provides a simple method to attempt quantification. This approach allows a straightforward estimation of dose effects, complementing the assessment of potential biological mechanisms contributing to treatment efficacy. We also acknowledge additional biological factors contributing to this treatment modality's efficacy. We proposed to increase dosage, focusing on standardizing the technique and analyzing clinical outcomes. We will examine potential protocol variations, primarily dose escalation. The literature suggests the primary mechanism of action in SFRT stems from changes activated by heterogeneous treatment administration. The intense dose neutralizes the tumor and induces stromal changes, involving immune system alterations that contribute to a distinctive response.

The limitations of the present study include its retrospective design, single-institution setting, small sample size, limited follow-up, and heterogeneity of tumor types and locations treated. In addition, the optimal timing of 2SG-RT relative to subsequent EBRT is not yet determined, and no comparator group or quantitative benchmarking against LRT or GRID techniques was included, limiting objective comparison of outcomes. Furthermore, the biological mechanisms implicated remain speculative, as the same concept applied in LRT has been adopted; although this is unsupported by direct biological correlates, the nominal doses administered clearly do not fully explain the notable outcomes observed. Additional limitations include the potential for selection bias and the lack of standardized patient-reported outcome metrics, which limits the objective assessment of palliative benefit. Despite these constraints, the positive preliminary clinical outcomes irrespective of tumor type or location suggest the technique's effectiveness. The aim is to assess initial clinical results, with the potential for designing a controlled study for further stratification by pathology.

Dose prescription in the heterogeneous fraction has been standardized for recent patients, with a more rigorous protocol developed. Long-term follow-up data collection is ongoing to analyze potential late toxicities and sustained responses. The criteria for adaptation of treatment are highly individualized, determined by an interdisciplinary tumor board, and based primarily on the percentage of tumor volume reduction observed in the follow-up CT scan performed for all patients.

The technique proposed in this work is not strictly an alternative to the well-established LRT and PATHY techniques. With respect to LRT, it does not consist of a grid of hot spheres and valleys, and with respect to PATHY, 2SG-RT is not limited to a portion of the PTV. 2SG-RT always involves the PTV to be treated in today's well-established techniques, in addition to a gradual intensification in the first session towards the volume of the highest tumor concentration. In our modest opinion, the advantage of this technique lies in its natural incorporation into routine clinical radiotherapy practice, allowing efficient implementation in settings where multiple patients must be treated daily. Furthermore, 2SG-RT is applicable to a wide variety of clinical situations and tumor volumes, without implying superiority over conventional LRT or other SFRT techniques.

The approach presented in this work is effective and represents an affordable, easily implementable alternative for patients with complex bulky tumors. 2SG-RT demonstrates adaptability to diverse tumor characteristics, including size, volume, location, and biological/metabolic properties, while facilitating expedited planning and delivery.

## Conclusions

Despite the multiple limitations of this study, the results lead us to believe that the developed 2SG-RT technique represents a feasible, efficient and promising approach to SFRT, applicable across a broad range of clinical scenarios, with enhanced computational and verification efficiency, and overall time-effectiveness. Furthermore, it offers the advantage of greater adaptability to physical, geometrical, and biological properties of tumors. This methodology constitutes a straightforward yet efficacious strategy, potentially facilitating subsequent radiotherapy or systemic treatment in patients who might otherwise be limited to palliative care. Given the preliminary nature of these findings, this work primarily serves to generate hypotheses and inform future research. EUD calculation and clinical results are consistent with the possibility that immune-mediated effects contribute to the response, which should be investigated in future studies. Further comparative studies with longer follow-up are warranted to validate these initial observations and clarify the clinical impact of 2SG-RT.
